# *Stomiosphaerina bakae* sp. nov., a new calcareous dinocyst of the Upper Cretaceous of the Central European Basin

**DOI:** 10.1371/journal.pone.0292531

**Published:** 2023-10-10

**Authors:** Agnieszka Ciurej

**Affiliations:** Department of Geology and Palaeontology, Institute of Biology and Earth Sciences, Pedagogical University of Krakow, Kraków, Poland; Balochistan University of Information Technology Engineering and Management Sciences, PAKISTAN

## Abstract

In this work the new species of *Stomiosphaerina bakae* sp. nov. is described. This is the third species of the genus *Stomiosphaerina*. Several dozen specimens of the newly described species were found within the Upper Cretaceous (upper Turonian) white chalk at Dubivtsi section near Halych in western Ukraine (south-eastern margin of Central European Basin). *Stomiosphaerina bakae* sp. nov. has an asymmetrical oval (pear-like shape) test, as it is wider in one side, and sharp in the opposite side, ranging from 55 to 67 μm in length and from 42 to 56.5 μm in width. It has a two-layered calcareous test. The outer layer, with thickness ranging from 4.5 to 6.5 μm, is built of long and wide plate-shaped calcite crystals and is white in plain-polarized light with a dark cross in crossed-polarized light. The inner layer, with thickness ranging from 1.5 to 4.5 μm, is yellowish, goldish to brownish color in plain-polarized light, with proximal and distal side smooth and even, and is built of short fibrous calcite crystals, without preferential orientation. One narrow (6 to 8 μm width) aperture is observed. *Stomiosphaerina bakae* sp. nov. differs from other species of S*tomiosphaerina*, i.e. S*tomiosphaerina biedai* Nowak 1974 and S*tomiosphaerina proxima* Řehánek 1987, by its cyst-shape, cyst-size, layers thickness, structure of outer layer and width of aperture. *Stomiosphaerina bakae* sp. nov. most likely corresponds to the non-defined species of S*tomiosphaerina* sp. described by Nowak 1974.

## Introduction

Calcareous dinoflagellate cysts or calcareous dinocysts are single-celled spherical calcareous microorganism, belonging to the Order Peridiniales, Class Dinophyceae. These calcareous structures are believed to be cysts formed during dinoflagellates life cycle stages (i.e. reproductive or resting stages [1–5] or to be skeletons of dinoflagellates with a vegetative coccid life stage [6–8]. About 30 extant species of calcareous dinoflagellates [2, 9–11] and about 260 fossil species (morphotypes) [e.g. 5, 12–17] have been described so far. The oldest cysts have been found in Upper Triassic deposits [18]. During the Cretaceous and Paleogene, this group reached its greatest species diversity [9, 19].

The genus *Stomiosphaerina* has been provided to represent dinoflagellate calcareous cysts in resting, reproductive or coccoid stages of their life cycle [20]. The genus *Stomiosphaerina* was introduced by Nowak [14] for unicellular, oval-shaped microfossils observed in thin sections of rocks. These forms have a calcareous test with a two-layered wall: the outer layer with radially arranged calcite crystals, with dark cross in crossed-polarized light, which is white in plain-polarized light, and the inner layer which is dark in plain-polarized light and aphanitic. The structural and optical features of specimens of the genus *Stomiosphaerina* are a combination of *Stomiosphaera-*like wall and *Cadosina-*like wall. One aperture is observed. Based on examinations of several dozen of specimens, Nowak [14] distinguishes new species of the genus *Stomiosphaerina*, named as a *Stomiosphaerina biedai*. Additionally, this author noted the presence of one taxon with features which could indicate a new species belonging to the genus *Stomiosphaerina*, however due to the small amount of material, he did not distinguish a new species. Both, *Stomiosphaerina biedai and Stomiosphaerina* sp. were found in “Żegocina Marls” of Turonian-?Santonian pelagic deposits of the Tethys deposits. These deposits was described in the Polish Outer Carpathians, near Bochnia in Żegociński Creek, below the dam, near the local church in Żegocina village, southern part of Poland. Later, Řehánek [15] created of another new species of the genus *Stomiosphaerina*, named *Stomiosphaerina proxima* Řehánek 1987. This species was found and described from greenish-grey, marly, microorganogenic lime mudstone of lower Berriasian (*Calpionella* zone) of Tethys deposits. This species was described in Central West Carpathian Paleogene basal breccias, in Lipany village in the eastern part of Slovakia. It was found in borehole Lipany 5, core No. 8, depth 2500–2504 m.

*Stomiosphaerina* species are typically known from pelagic deposits from Tethys localities, however, Olszewska et al. [21] described *Stomiosphaerina biedai* Nowak 1974 in Upper Cretaceous (Turonian) epicontinental sediments in southern Poland and south-western Ukraine. This finding widened the paleogeographical distribution of the genus *Stomiosphaerina* to the epicontinental deposits in the Central European Basin. Recently, *Stomiosphaerina biedai* Nowak 1974 was also described in the upper Turonian deposits of the south-eastern margin part of the Central European Basin, from the Dubivtsi section in western Ukraine [22].

In this paper, a new species of this genus, *Stomiosphaerina bakae* sp. nov., is described. This taxon is abundant in the upper Turonian deposits within the Dubivtsi Formation from the Dubivtsi section in western Ukraine (the south-eastern part of the Central European Basin*)*. The taxon was distinguished based on the examinations of several dozen specimens in various cross sections, observed under an optical microscope and scanning electron microscopy (SEM). Forty specimens were biometrically measured.

### Material

**Fig 1:**
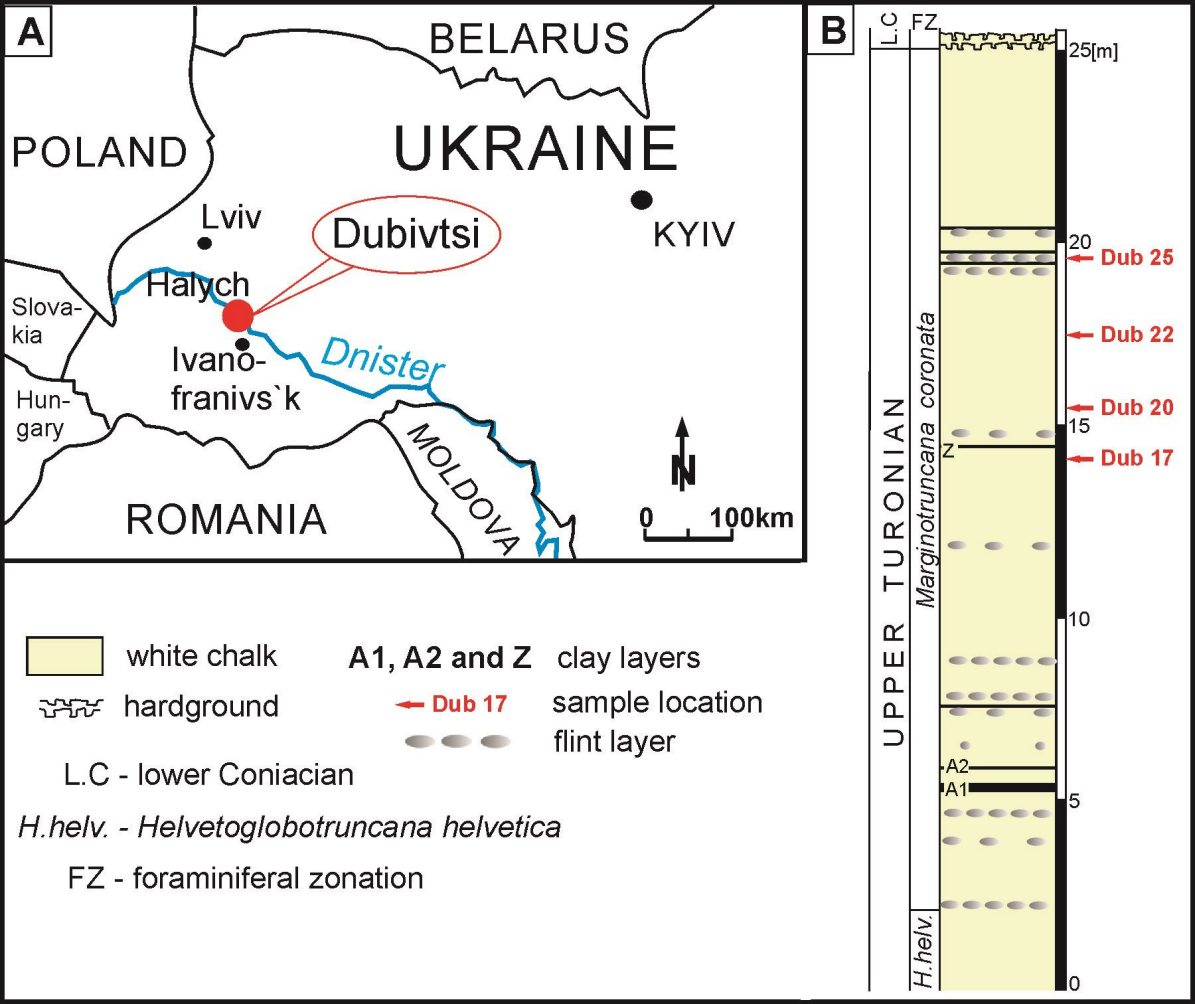
Location of the Dubivtsi section in western Ukraine. A–General geographic location marked by red circle, modified after [33], **B**–Lithological column at Dubivtsi section as logged in 2009, after [33], slightly modified. Foraminifera zones according to Dubicka and Peryt [33] and Walaszczyk and Peryt [36].

The material studied was obtained from the Dubivtsi section in western Ukraine (Fig 1A). Geologically, it is situated in the south-western margin of the East European Platform, within the Lviv-Stryi Syncline, which belongs to the bigger tectonic unit, called the Border Synklinorium [23–25]. In the Volyno to Podolye area of the western Ukraine, Turonian and Coniacian deposits are detected [e.g. 26–28]. These deposits belong to the lithological unit referred here as the Dubivtsi Formation and originally named as “Dubivtsi Suite” [26–28]. The total thickness of this formation ranges from 54 to 158 m. It is divided into two units: (1) the lower unit of Turonian age, composed of the white and grey limestones, with abundant calcareous dinocysts of *Pithonella* genus [29–33] and flint concretions that appear 15–20 m above the base of the unit, and increase upward in the section [28, 34, 35]; and (2) the upper unit which is Coniacian age and containing limestones, often argillaceous and marls with common fragments of inoceramid [28].

The Dubivtsi section is located in an abandoned quarry, 1 km to the east of Dubivtsi village, and to the south of Halych town. The studied section contains four lithological units of the Cretaceous deposits of the Dubivtsi Formation, as follows from bottom to top: 1) white chalk (25 m thick, strongly lithified, with CaCO_3_ content ranging from 97.8 to 99.9%), 2) hardgrounds, 3) inoceramid limestones (4.4 m thick), and 4) marls (1.5 m thick). The total thickness of the section is 31 m. For a detailed profile see Dubicka and Peryt [33]. According to the biostratigraphical division, white chalk corresponds to the upper Turonian, and inoceramid limestones and marls represent the Coniacian [33]. The lower part of the section belongs to the *Helvetoglobotruncana helvetica* Zone, the middle part to the *Marginotruncana coronata* Zones, and the uppermost part (inoceramid limestones and marls) corresponds to the *Marginotruncana sinuosa* Zone [33, 36].

**Fig 2:**
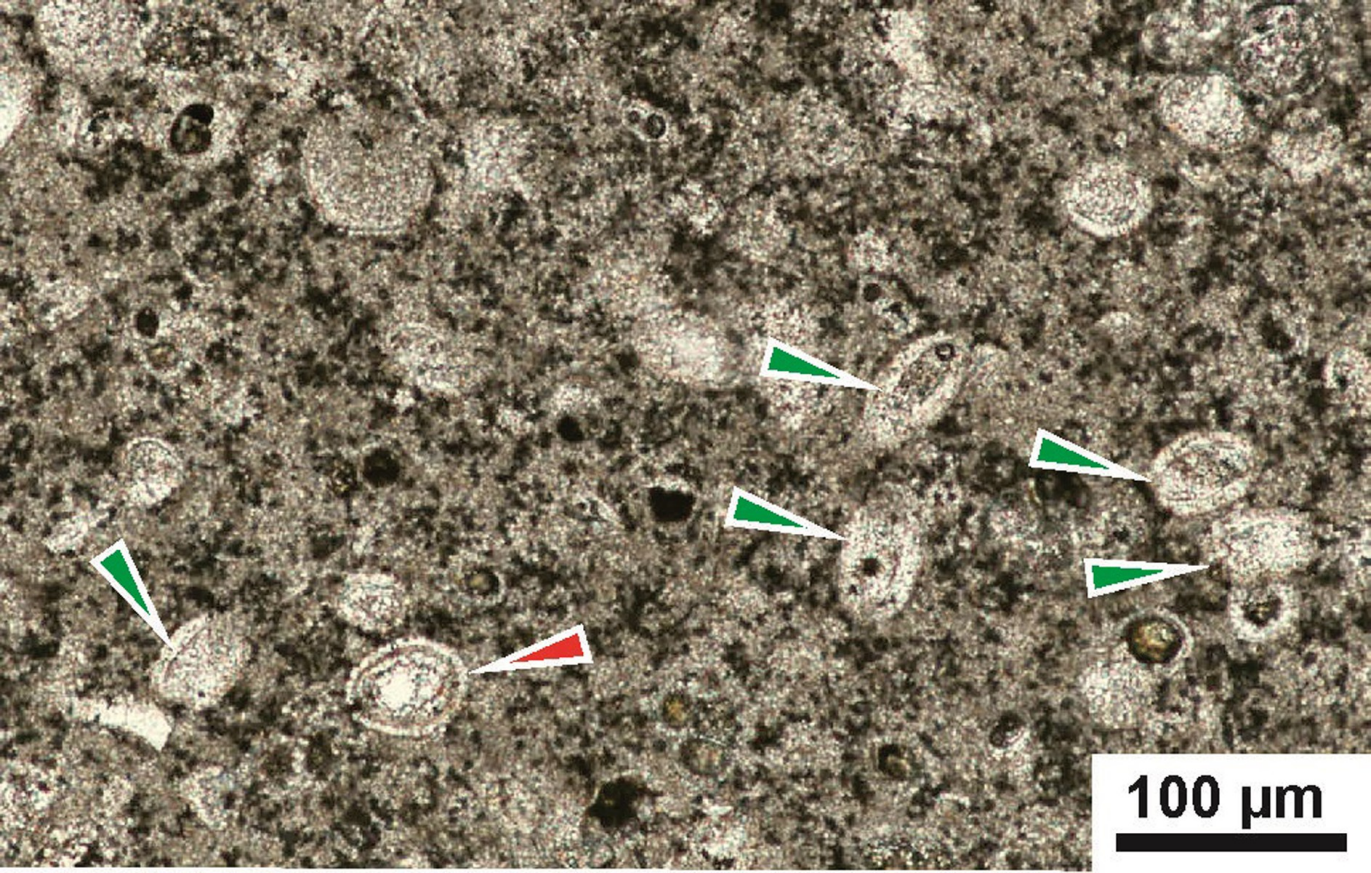
Microscopic view of wackestone/packstone with *Stopmiosphaerina bakae* sp. nov. *Stopmiosphaerina bakae* sp. nov. (red arrow) is accompanied by common *Pithonella ovalis* (Kaufmann in Heer 1865) Lorenz 1902 (green arrows). Thin section, plain-polarized light, Sample Dub 17.

The material used in this study was obtained from the white chalk of the Dubivtsi Fm. The samples were originally collected in 2009 by Zofia Dubicka (Warsaw University, Warszawa, Poland). These samples were a subset of the 32 samples of white chalk that were studied for foraminifera by Dubicka and Peryt [33]. Calcareous dinocysts were studied in 16 samples of the white chalk. The newly defined *Stomiosphaerina bakae* sp. nov. has been found in four samples: Dub 17, Dub 20, Dub 22 and Dub 25, which were taken from the middle and upper part of the chalk succession in Dubivtsi section (Fig 1B). These rock samples, described as wackestone/packstone, contain several dozen specimens of the newly defined *Stomiosphaerina bakae* sp. nov. (Fig 2). Other calcareous dinocysts present in the studied samples are: *Pithonella ovalis* (Kaufmann in Heer 1865) Lorenz 1902 (Fig 2) and *Pithonella sphaerica* (Kaufmann in Heer 1865) Zügel 1994, which are very common, and *Pithonella lamellata* Keupp in Keupp & Kienel 1994 and *Pithonella cardiiformis* Zügel 1994 represented in less abundance. Specimens of *Bonetocardiella conoidea* Bonet 1956 and *Stomiosphaerina biedai* Nowak 1974 were also detected. Detailed analysis of calcareous dinocysts from this section is the subject of a separate publication by Ciurej and Dubicka [22]. The co-occurrence of *S*. *biedai* and *P*. *cardiiformis* indicates the upper Turonian for studied white chalk [22], what is in accordance with foraminiferal biostratigraphy by Dubicka and Peryt [33].

## Methods and sample storage

The newly described species of calcareous dinocyst—*Stomiosphaerina bakae* sp. nov., has been distinguished based on the examinations of several dozen specimens in various cross sections observed in four samples: Dub 17, Dub 20, Dub 22 and Dub 25. Forty specimens were biometrically measured.

Calcareous dinocysts were observed in thin sections of the rocks of 3x5 cm size. The observations were made under a Nikon Eclipse LV100N POL polarizing optical microscope with a digital camera and NIS-Elements BR software (Department of Geology, Pedagogical University of Krakow). The following parameters of the microscope instrumentation settings were used, including various light parameters: (a) plane-polarized light, (b) crossed-polarized light, and (c) reflected light. Calcareous dinocysts were also observed in the rock chops under scanning electron microscopy (SEM) at HITACHI 3–4700 housed at the Laboratory with Field Scanning Emission Microscopy and Microanalysis at the Institute of Geological Sciences of the Jagiellonian University, Krakow, Poland. The broken surface (without any chemical treatment) of the rock chips, in size 2x3 cm, was coated by gold and observed under secondary electron (SE) mode, with acceleration voltage set at 20 keV on a high vacuum, and work distance of approximately 13.0 mm (12.4 mm to 14.4 mm).

The digging and site access permits of these rock samples were not required for this study. There are no legal or ethical restrictions being placed upon the data.

The holotype and paratypes, designed in thin section no. Dub 17, are deposited in the collections of the European Micropaleontological Reference Centre (EMRC), Address: Micropress Europe al. Mickiewicza 30; 30–059 Krakow, Poland, email: info@micropresseurope.eu and housed in Cabinet 7, drawer 11. Collection reference is EMRC 7/11. The repository number Dub 17, thin section.

## Results and discussion

### Systematic palaeontology

Domain **Eukaryota** Chatton 1925

Kingdom **Chromista** Caval.-Sm. (1981)

Subkingdom **Harosa** Cavalier-Smith 2010

Infrakingdom **Alveolata** Cavalier-Smith 1991

Phylum **Miozoa** Cavalier-Smith 1987

Subphylum **Myzozoa** Cavalier-Smith and Chao 2004

Infraphylum **Dinozoa** Cavalier-Smith 1981

Superclass **Dinoflagellata** (Bütschli, 1885) Fensome et al. 1993

Class **Dinophyceae** Pascher 1914

Subclass **Peridiniphycidae** Fensome et al. 1993

Order **Peridiniales**, Haeckel 1894

Family **Thoracosphaeraceae** Schiller 1930

Genus ***Stomiosphaerina*** Nowak 1974

**Species:** *Stomiosphaerina bakae* sp. nov.

(Figs 3–7)

**Fig 3:**
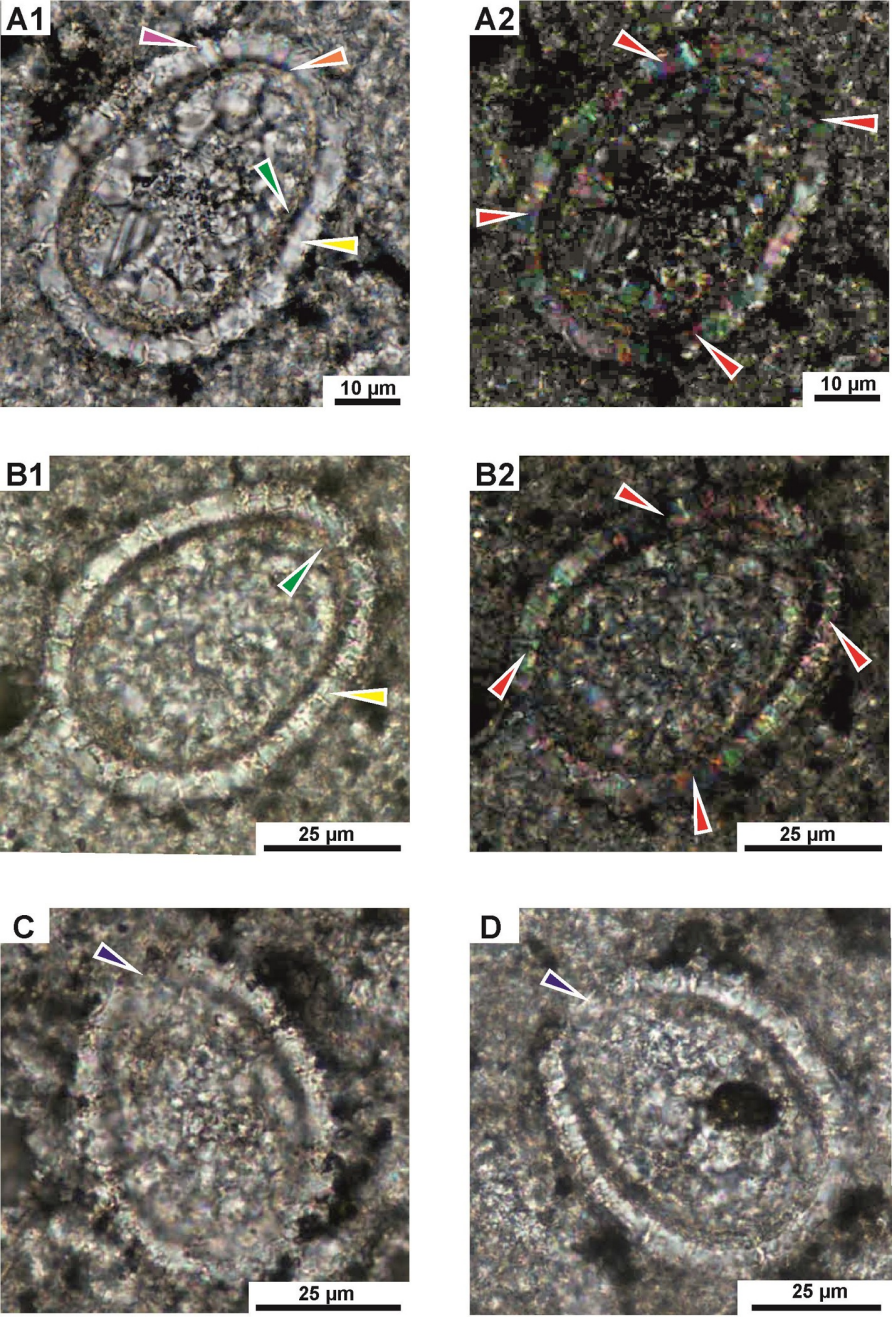
*Stomiosphaerina bakae* sp. nov. **A–**holotype—repository number: Dub 17-1-H seen in longitudinal section of a test, specimen number 1 in Table 1. **A1** –asymmetrical oval shape (pear-like shape) and morphology of two layers: inner (green arrow), which is thin and even, observed as a brownish color and outer layer (yellow arrow) composed of the coarse, wide and long plate-shaped calcite crystals. The proximal margin of the outer layer (pink arrow) is distinct and observed as a characteristic rim formed by distinct pyramidal top of calcite crystals. Note the smooth and even distal margin of the outer layer (orange arrow). The distinct boundary between the outer and inner layer is clearly visible. **A2 –**dark cross (red arrows) is clearly visible. **B**–longitudinal section of specimen number 2 in Table 1: **B1 –**clearly visible outer layer with coarse calcite crystals (yellow arrow), and dark, brownish color of inner layer with fibrous calcite (green arrow). **B2 –**dark cross (marked by red arrows) is visible. **C**–paratype 1—repository number: Dub 17-3-P1, specimen number 3 in Table 1. Longitudinal section of the test with narrow aperture (blue arrow). **D–**longitudinal section of specimen number 4 in Table 1 showing test with hardly visible aperture (blue arrow). Sample Dub 17. Thin section. A1, B1,C,D–plain-polarized light; A2,B2 –crossed-polarized light.

**Fig 4:**
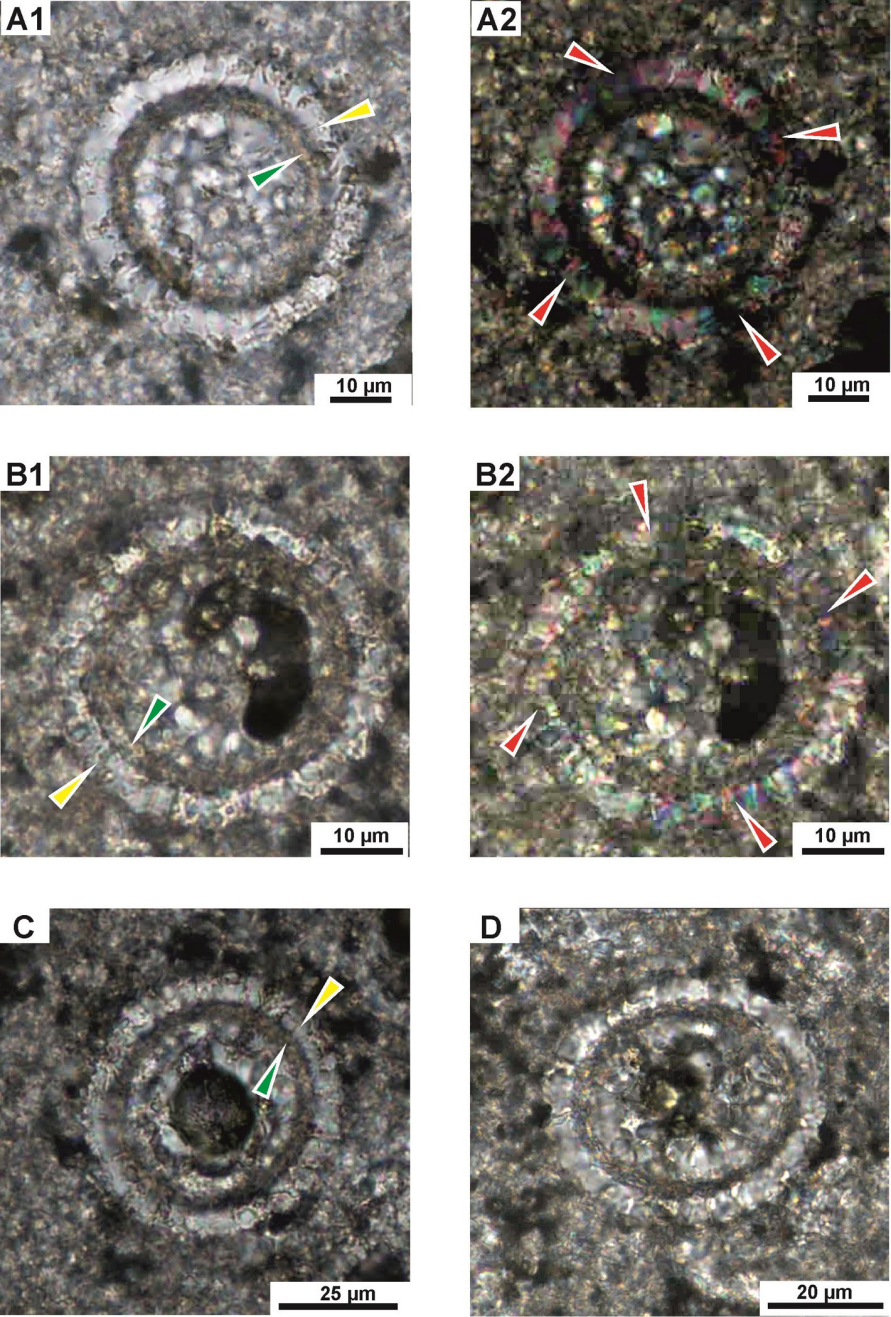
*Stomiosphaerina bakae* sp. nov. **in transversal sections. A**–paratype 2—repository number: Dub 17-5-P2, specimen number 5 in Table 1. **A1** –circular shape of a cyst with well-preserved thin inner (green arrow) and thicker outer (yellow arrow) layers. Note the equal thickness of the inner layer. **A2 –**dark cross (red arrows) is clearly visible. **B–**specimen number 6 in Table 1. **B1 –**small cyst with hardy recognizable inner layer. **B2** –note the dark cross (red arrows). **C,D–**specimens number 7 and 8, respectively in Table 1, showing a circular shape of a cyst with well visible, thick inner layer and hardly recognizable outer layer from the surrounding material. Sample Dub 17. Thin section. A1,B1,C,D–plain-polarized light; A2,B2 –crossed-polarized light.

**Fig 5:**
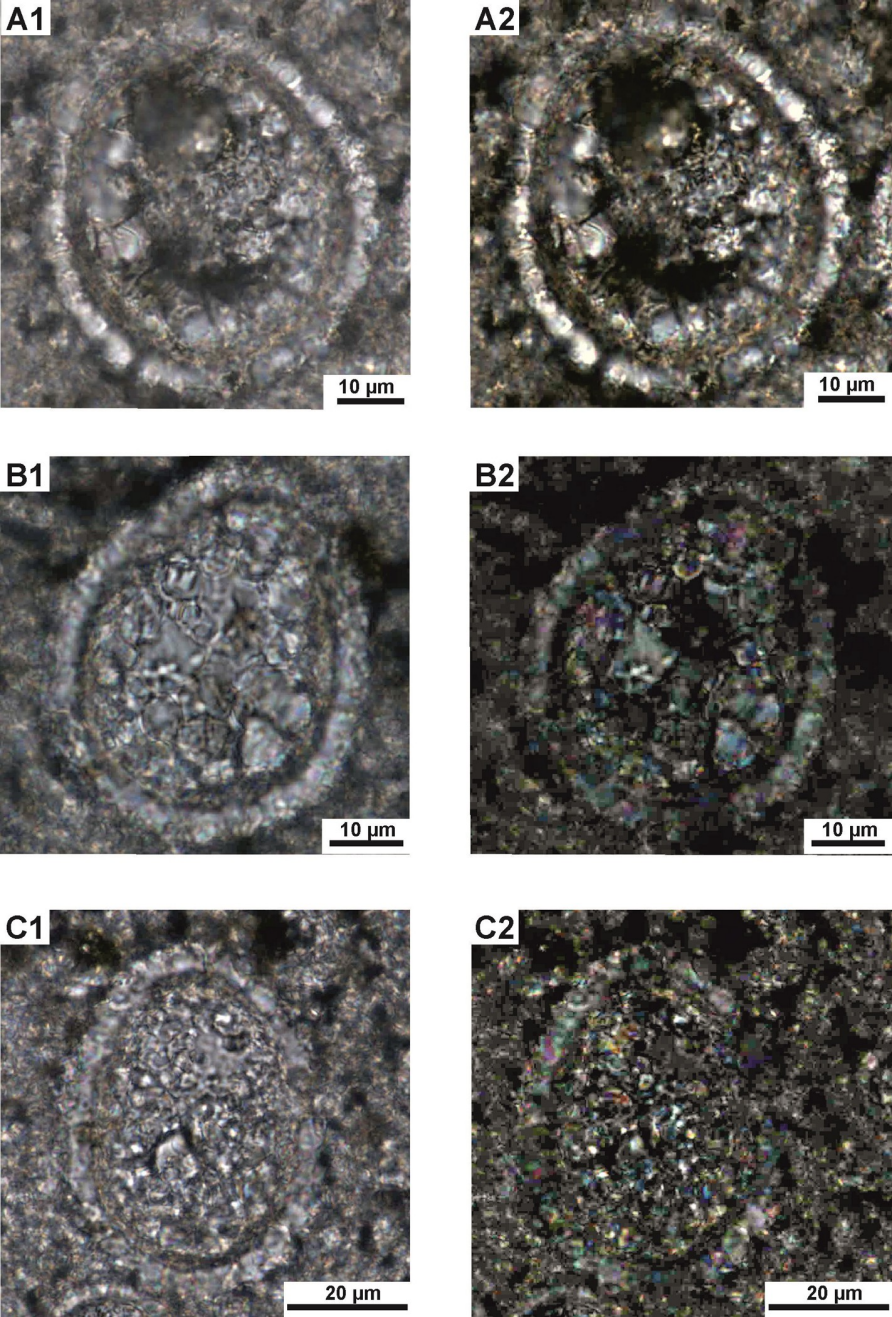
*Stomiosphaerina bakae* sp. nov. in longitudinal sections in various stage of preservation. **A,B**–specimens number 9 and 10, respectively in Table 1, **A1,B1** –well visible asymmetrical oval test in moderate state of preservation. **A2,B2** –dark cross is hardly visible. **C–**section of specimen number 11 in Table 1. **C1**—specimen is hardly recognizable from the surrounding material; the inner layer is hardly visible because of advance diagenesis. **C2** –dark cross is hardly visible. Sample Dub 17. Thin section. A1,B1,C1 –plain-polarized light; A2,B2,C2 –crossed-polarized light.

**Fig 6:**
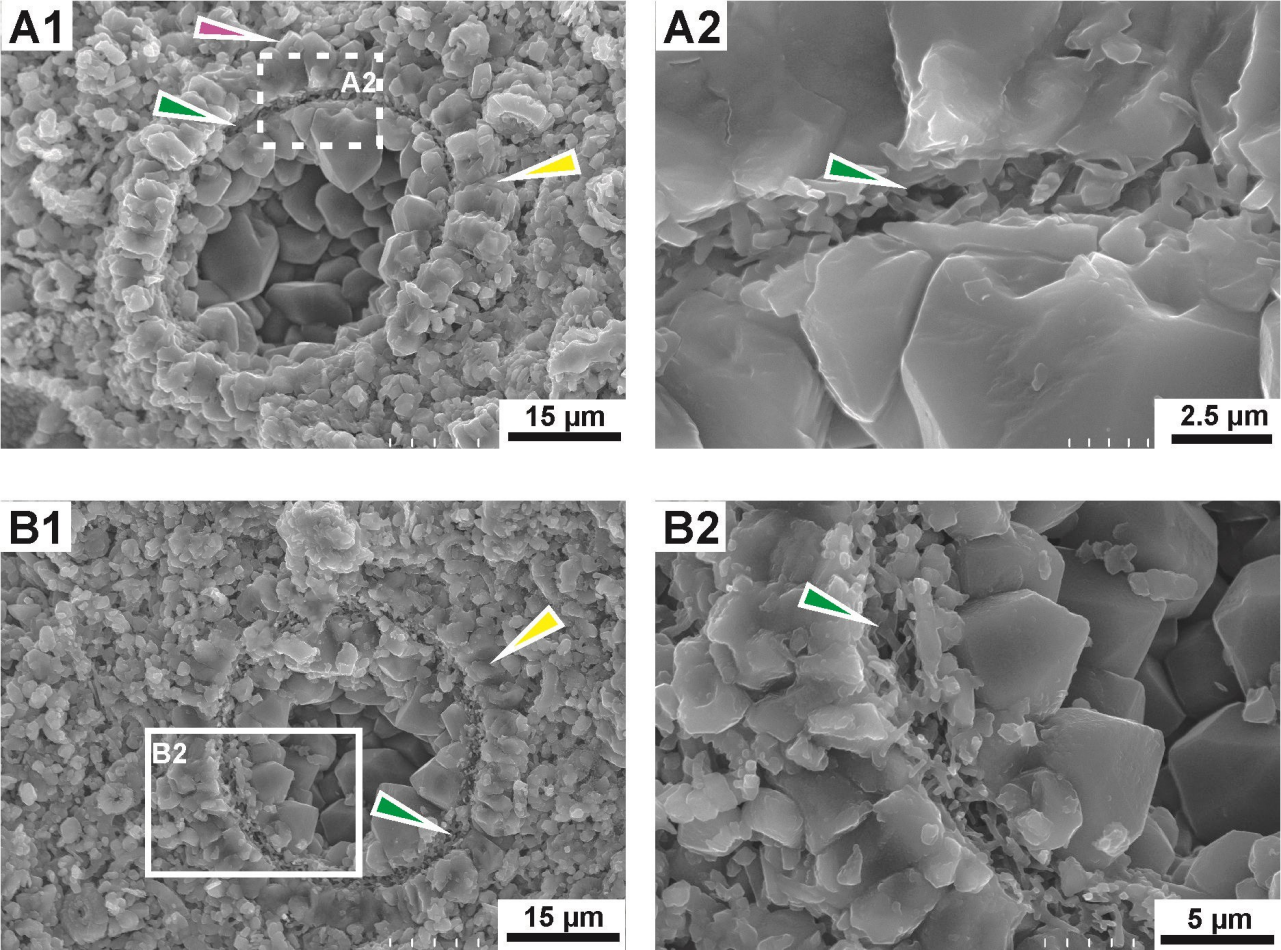
Scanning electron microscope photomicrograph of transversal sections of *Stomiosphaerina bakae* sp. nov. **cyst. A1,B1 –**showing the structure of the wall, composed of thick outer (yellow arrow) and thinner inner (green arrow) layers. The outer layer is composed of coarse, long and wide, regular plate-shaped calcite crystals, radially arranged to the cyst surface; note calcite plate-like (tabular) crystals with a distinct, pyramid-like top (pink arrow) on a proximal margin of the outer layer. The inner layer is composed of thin, short, fibrous calcite crystals, without preferential orientation to the cyst surface, enlarged in A2 and B2. **A2,B2** –enlarged images from A1 and B1, respectively, showing details of inner layer with chaotically arranged, thin, short, fibrous calcite crystals (green arrows). Rock chips. Dub 17.

**Fig 7:**
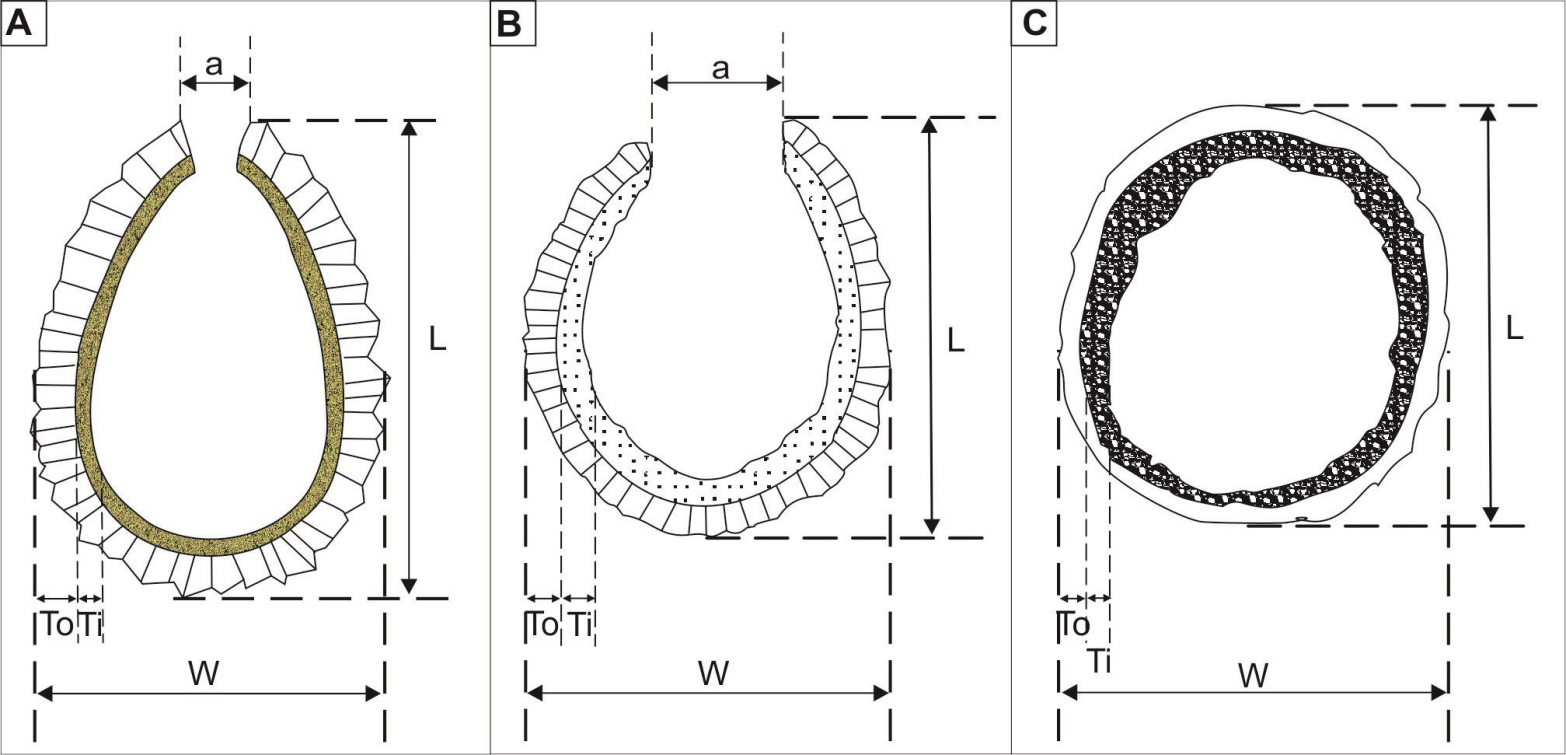
Schematized drawing of test with two layers (outer and inner) of cysts of genus *Stomiosphaerina*. **A**—*Stomiosphaerina bakae* sp. nov. (based on holotype and paratype 1 cross-sections), **B**—*Stomiosphaerina biedai* Nowak 1974, (redrawn from Nowak [14]), **C—***Stomiosphaerina proxima* Řehánek 1987 (redrawn from Řehánek [15]). Not to scale. L–length of test, W–width of test, To–thickness of outer layer, Ti–thickness of inner layer, a–width of aperture.

#### Derivatio nominis

The species is dedicated to Palaeontologist Marta Bąk (AGH, University of Science and Technology in Krakow, Poland).

#### Diagnosis

shape of test and chamber is asymmetrical oval, as it is wider in one side and sharper in the opposite side (pear-like shape), double-layered wall, thick outer layer, composed of coarse, wide and long plate-shaped calcite crystals with dark cross in crossed-polarized light, and thin inner layer, composed of short, fibrous calcite crystals. One narrow aperture.

#### Description

Test and chamber of the cyst in longitudinal section with an asymmetrical oval shape: wider on one side and sharper on the opposite side (pear-like shape). Transversal section is circular, both cyst and chamber. The chamber is located centrally. One narrow aperture is situated in a sharper side of the cyst, however, often it is hardly visible (Fig 3C and 3D). The calcareous wall is mostly uniform in thickness and is composed of two distinctly separated layers: inner and outer (Figs 3–7A).

The outer layer is composed of the coarse, wide and long plate-like calcite crystals, which show a rather regular shape and with sizes of about 2.5 to 6 μm wide and 4.5 to 6.5 long, and c-axis arranged radially to cyst surface (Fig 6). The outer layer in plain-polarized light has a milky-white color. In cross-polarized light this layer displays the dark cross, which is related with the optical orientation of the calcite crystals in relation to the main cross sections of the polarizers in a polarizing optical microscope (Figs 3–5). The thickness of this wall is approximately equal in both longitudinal and transverse sections. Proximal margin (proximal side) of the outer layer is often developed as a characteristic rim formed by distinct pyramidal top of calcite crystals (Fig 6A1, see pink arrow). However, sometimes, this proximal side can be highly ragged and uneven because of advanced recrystallization of calcite crystals. The distal margin (distal side) of the outer layer is smooth and even. The boundary between the outer and inner layer is clearly visible.

The inner layer is built of short, thin, fibrous, calcite crystals, without preferential orientation to the cyst surface (Fig 6). In plain-polarized light this layer is yellowish, golden, to brownish colored. In polarized light the dark cross is not visible. The thickness is equal, regardless of the cross section of the cyst, however the inner layer sometimes shows minimal thinning in the sharper side of the cyst. The proximal and distal side of the inner layer is smooth and even, without irregularities.

#### Dimensions

**Fig 8:**
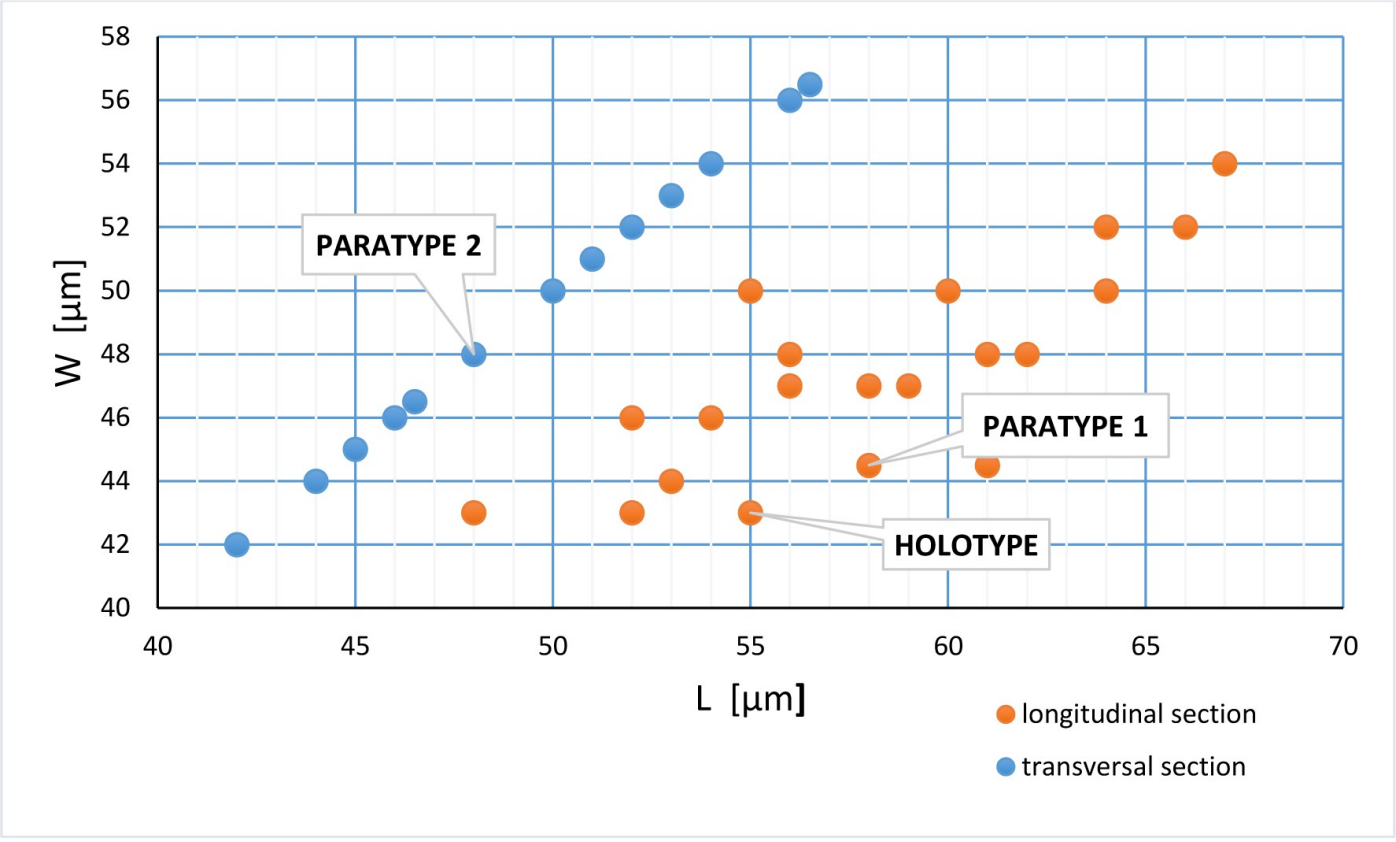
*Stomiosphaerina bakae* sp. nov. diagram showing the relation between the length (L) and width (W), based on data from Table 1.

**Fig 9:**
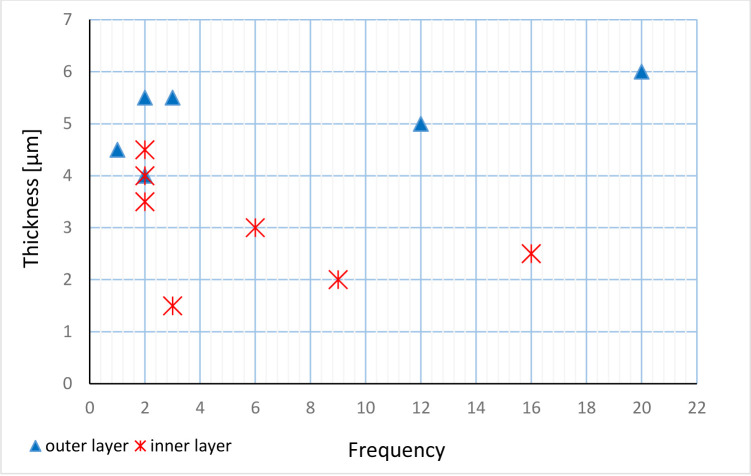
Thickness frequency of individual layers in the wall of the species cysts *Stomiosphaerina bakae* sp. nov.

**Fig 10:**
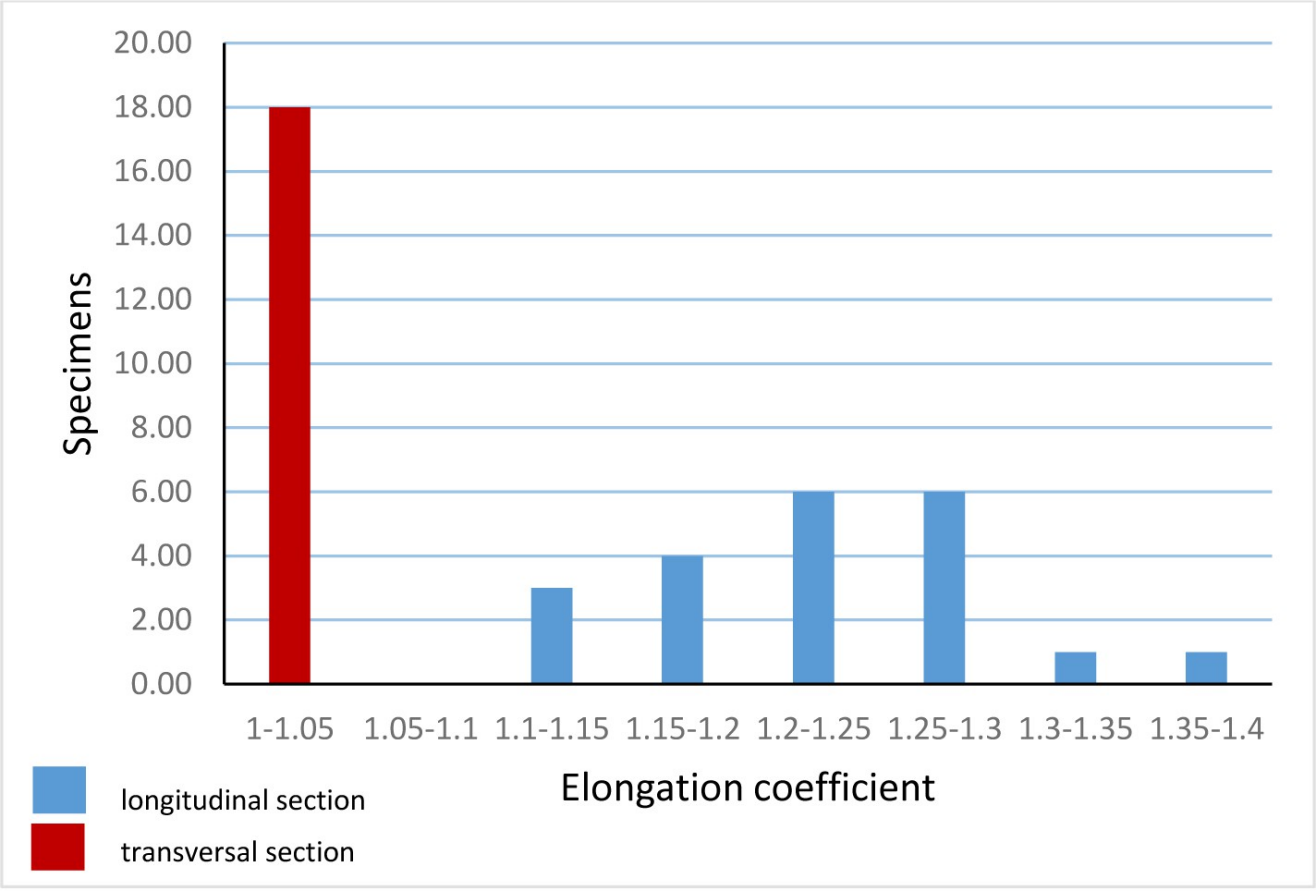
*Stomiosphaerina bakae* sp. nov. diagram showing the elongation coefficient (Ec = L/W).

The parameters of studied specimens are presented in Table 1. The length of the test varies from 55 to 67 μm and width varies from 42 to 56.5 μm (Fig 8). The outer layer is thicker than the inner layer. The thickness of the outer layer range from 4.5 to 6.5 μm. The thickness of the inner layer range from 1.5 to 4.5 μm. Thickness frequency of individual layers in the wall of the species is shown in Fig 9. The outer and inner layers thickness ratio ranges from 1.1:1 up to 3:1. Aperture width range from 6 to 8 μm (Fig 3C and 3D). The elongation coefficient (Ec) was calculated as the ratio of the length (L) to the width (W) of the cyst measured for the 40 specimens (Fig 10). This parameter for specimens in transversal section ranges from 1.00 to 1.05 and for those in longitudinal section varies from 1.10 to 1.37. The most abundant values for specimens in longitudinal section are within the range of 1.2 to1.3.

**Table 1:** Dimensions of the specimens of *Stomiosphaerina bakae* sp. nov. from samples no. Dub 17, Dub 20, Dub 22 and Dub 25.

Specimen number	Location	Cyst section	L [μm]	W [μm]	To [μm]	Ti [μm]	L/W	To/Ti	Diameter of aperture [μm]	Remarks	Repository number	Figure
**1**	**Dub 17**	**Long**	**55**	**43**	**6**	**2.5**	**1.28**	**2.40**		**HOLOTYPE**	**Dub 17-1-H**	**3A**
2	Dub 17	Long	66	52	6	2.5	1.27	2.40				3B
**3**	**Dub 17**	**Long**	**58**	**44.5**	**6**	**2.5**	**1.30**	**2.40**	**7**	**PARATYPE 1**	**Dub 17-3-P1**	**3C**
4	Dub 17	Long	64	52	5.5	2.5	1.23	2.20	8			3D
**5**	**Dub 17**	**Trans**	**48**	**48**	**6**	**2.5**	**1.00**	**2.40**		**PARATYPE 2**	**Dub 17–5-P2**	**4A**
6	Dub 17	Trans	42	42	5	2	1.00	2.50				4B
7	Dub 17	Trans	52	52	6	4	1.00	1.50				4C
8	Dub 17	Trans	50	50	5	3	1.00	1.66				4D
9	Dub 17	Long	55	50	5	2.5	1.10	2.00				5A
10	Dub 17	Long	54	46	5	2.5	1.17	2.00				5B
11	Dub 17	Long	61	44.5	5.5	2.5	1.37	2.20				5C
12	Dub 17	Long	64	50	6	3	1.28	2.00				
13	Dub 17	Long	56	48	5.5	2.5	1.17	2.20				
14	Dub 17	Long	60	50	6	2.5	1.20	2.40				
15	Dub 17	Long	56	47	5	2	1.19	2.50				
16	Dub 17	Long	67	54	6.5	3	1.24	2.17	8			
17	Dub 17	Trans	56.5	56.5	6	3	1.00	2.00				
18	Dub 17	Trans	54	54	6	2	1.00	3.00				
19	Dub 17	Trans	46	46	5	1.5	1.00	3.33				
20	Dub 17	Trans	45	45	4	1.5	1.00	2.67				
21	Dub 17	Trans	48	48	5	2	1.00	2.50				
22	Dub 17	Trans	56	56	6	2.5	1.00	2.40				
23	Dub 17	Trans	51	51	6	2	1.00	3.00				
24	Dub 17	Trans	50	50	6	2.5	1.00	2.40				
25	Dub 17	Trans	52	52	6	3	1.00	2.00				
26	Dub 17	Trans	50	50	6	2	1.00	3.00				
27	Dub 20	Long	53	44	5	4	1.20	1.25				
28	Dub 20	Long	52	46	6	2.5	1.13	2.40				
29	Dub 20	Long	59	47	6.5	3.5	1.26	1.86				
30	Dub 20	Long	52	43	5	2.5	1.21	2.00				
31	Dub 22	Long	56	48	6	3	1.17	2.00	7			
33	Dub 22	Long	58	47	5	2.5	1.23	2.00				
34	Dub 22	Long	62	48	6	4.5	1.29	1.33				
35	Dub 22	Trans	44	44	4.5	2	1.00	2.25				
36	Dub 22	Trans	53	53	4	1.5	1.00	2.67				
37	Dub 25	Long	48	43	5	4.5	1.12	1.11				
38	Dub 25	Long	61	48	6	3.5	1.27	1.71				
39	Dub 25	Trans	46.5	46.5	5	2	1.00	2.50				
40	Dub 25	Trans	50	50	6	2	1.00	3.00				

L—length; W -width; TO—thickness of outer layer, TI—thickness of inner layer, Long—longitudinal section, Trans—transversal section

**Holotype:** species shown in Fig 3A, repository number Dub 17-1-H, thin section no. Dub 17, collection reference is EMRC 7/11, located in the European Micropaleontological Reference Centre (EMRC), Cabinet 7, drawer 11. Longitudinal section, dimensions: length of the test 55 μm, width is 43 μm, thickness of the outer layer: 6 μm, thickness of the inner layer 2.5: μm, elongation coefficient: 1.28, the outer and inner layers thickness ratio is 2.40.

**Paratype 1:** species shown on Fig 3C, repository number Dub 17-3-P1, thin section no. Dub 17, collection reference is EMRC 7/11, located in the European Micropaleontological Reference Centre (EMRC), Cabinet 7, drawer 11. Longitudinal section, dimensions: length of the test 58 μm, width is 44.5 μm, thickness of the outer layer: 6 μm, thickness of the inner layer: 2.5 μm, aperture width: 7 μm, elongation coefficient (Ec): 1.30, the outer and inner layers thickness ratio is 2.40.

**Paratype 2:** species figured in Fig 4A, repository number Dub 17-5-P2, thin section no. Dub 17, collection reference is EMRC 7/11, located in the European Micropaleontological Reference Centre (EMRC), Cabinet 7, drawer 11. Transversal section, dimensions: length and width of the test 48 μm, thickness of the outer layer: 6 μm, thickness of the inner layer: 2.5 μm, elongation coefficient (Ec): 1.00, the outer and inner layers thickness ratio is 2.40.

**Age (Biostratigraphical range):** late Turonian, *Marginotruncana coronata* Zone [33].

**Stratigraphic range:** upper Turonian white chalk of the Dubivtsi Formation.

**Locus typicus:** Dubivtsi, abandoned quarry in the western Ukraine, south-western part of the East European Platform.

**Material:** several dozen of specimens in various cross sections observed under an optical microscope and scanning electron microscope, including 40 specimens biometrically measured (Table 1).

**Diagnosis differentials:** *Stomiosphaerina biedai* Nowak 1974 differs from *S*. *bakae* sp. nov. by its markedly oval shape (symmetrical in both edges) of test and chamber in longitudinal section (Fig 7B). *Stomiosphaerina biedai* has slightly smaller tests, with length ranging from 39.5 to 60.5 μm and with width ranging from 34 to 51 μm (see comparison in Table 2). The structure of the outer wall of *S*. *biedai* differs by its thinner, and shorter, bladed-type calcite crystals occur. The outer layer of *S*. *biedai* is thinner (ranging from 2.6 to 5.2 μm). The inner layer can be thicker, as it ranges up to 5.26 μm. Cysts of *S*. *biedai* have a wider range of inner and outer layers thickness ratios (minimum 0.5:2 and maximum 2:1). Nowak [14], in studied Turonian material from Polish Outer Carpathians described three groups of *S*. *biedai* with differences in the inner and outer layer thickness ratio: (a) cysts with both layers with similar or the same thickness (about 3.95 μm), (dominant group), (b) cysts with a distinctly thicker inner layer than the outer layer (less abundant group) and (c) cysts with significantly thinner inner layer than the outer layer (the least numerous group). *Stomiosphaerina biedai* also differs by the presence of numerous irregularities of the inner side of the inner layer. The porosity described by Nowak [14] in the outer layer of S. *biedai* was not observed in *S*. *bakae* sp. nov. Species S. *biedai* also differs by a much wider aperture (from 11.8 to 15.78 μm width), as its diameter is 1/3–1/4 the width of the test.

**Table 2:** Comparison of dimensions of *Stomiosphaerina bakae* sp. nov., *Stomiosphaerina biedai* Nowak 1974, *Stomiosphaerina* sp. described by Nowak [14] and *Stomiosphaerina proxima* Řehánek 1987.

Species	No.	L [μm]	W [μm]	To [μm]	Ti [μm]	L/W	Cyst section	Data source
*Stomiosphaerina biedai*	1	42.08	34.19	2.63	3.69	1.23		Nowak [14]
	2	39.45	36.82	3.94	1.32	1.07		Nowak [14]
	3	49.97	42.08	3.94	3.95	1.18		Nowak [14]
	4	60.49	47.34	2.63	3.95	1.28		Nowak [14]
	5	57.86	49.97	3.94	3.95	1.15		Nowak [14]
	6	55.23	51.28	3.95	5.26	1.07		Nowak [14]
*Stomiosphaerina* sp.	7	50	40	3.94	3.94	1.26		Nowak [14]
*Stomiosphaerina proxima*	8	92	85.5	10	10	1.07		Řehánek [15]
	9	78.5	78.5	7	13.5	1,0		Řehánek [15]
	10	72	68.5	10	6.5	1.05		Řehánek [15]
	11	68.5	65	3.5	10	1.05		Řehánek [15]
	12	65	61.5	2	8.5	1.05		Řehánek [15]
	13	58	48	10	8.5	1.20		Řehánek [15]
*Stomiosphaerina bakae sp nov*.	14	42	42	5	2	1.00	Trans	this paper, sample: Dub 17
	15	48	48	5	2	1.00	Trans	this paper, sample: Dub 17
	16	54	46	5	2.5	1.17	Long	this paper, sample: Dub 17
	17	55	43	6	2.5	1.28	Long	this paper, sample: Dub 17
	18	56	48	5.5	2.5	1.17	Long	this paper, sample: Dub 17
	19	64	50	6	3	1.28	Long	this paper, sample: Dub 17
	20	67	54	6.5	3	1.24	Long	this paper, sample: Dub 17

L—lengh; W—width; To—thickness of outer layer, Ti—thickness of inner layer, Long—longitudinal, Trans—transversal

*Stomiosphaerina proxima* Řehánek 1987 differs from the newly described species by its circular shape and its bigger size (Fig 7C). The cyst length ranges from 58 to 92 μm and width ranges from 48 to 85.5 μm (see Table 2). The thickness of the layers is also higher. The thickness of the outer layer ranges from 2.0 to 10 μm. The thickness of the inner layers range from 5.0 to 13.5 μm. The outer layer differs by relatively fine-grained calcite crystals (a very fine “spherulitic texture”). However, this layer also displays a dark cross in crossed-polarized light. The inner layer differs by the presence of chaotic “porcelaneous texture”. However, this layer shows similarities in color as light-brown color in plain-polarized light is observed. The stratigraphic position of *S*. *proxima* species is also different, as it occurs in deposits of Berriasian age [15].

Similarities in morphology are observed in specimens described as *Stomiosphaerina* sp. by Nowak [14] from a similar stratigraphic position–Turonian-?Santonian [14]. The only one specimen described by Nowak [14] has a similar shape of the cyst: “Pear-shaped microfossil” and “It is wider in the upper part, with sharp closure in the lower part (like a caudal process).”, see Nowak [14, p. 56]. The cyst size of this taxon (50 μm length and 40 μm width (length/with ratio is 1.26)) is in the range of *S*. *bakae* sp. nov. The thickness of layers was not precisely described for this taxon, however, from measures made by author of recent paper, of *Stomiosphaerina* sp. from a figure by Nowak [14, Plate II, Figs 1 and 2], it can be assumed that the outer layer is thicker than the inner one, and the outer and inner layers thickness ratio is 1.2:1, which is in range of *S*. *bakae* sp. nov. Nowak [14, p. 56] observed “some tailing out of the inner layer in the upper most part of the test” in *Stomiosphaerina* sp. Some minimal thinning of the inner layer in the sharper side of the cyst of *S*. *bakae* sp. nov. can sometimes be observed. All these similarities suggests *Stomiosphaerina* sp. of Nowak [14] most likely corresponds to *Stomiosphaerina bakae* sp. nov.

## Conclusions

Upper Turonian white chalk deposits of the Dubivtsi Formation deposited in the shallow water environments of the south-eastern margin of Central European Basin [33, 35] contain numerous microfossils of *Stomiosphaerina bakae* sp. nov. Microscopic examinations by optical microscope and scanning electron microscope provide diagnostic criteria of *Stomiosphaerina bakae* sp. nov., that may be summarized as follows:

- an asymmetrical oval (pear-like shape) calcareous test, with length ranging from 55 to 67 μm and width from 42 to 56.5 μm,
- pear-like chamber, located in the central part of the cyst,
- wall composed of two layers,
- outer layer, with thickness from 4.5 to 6.5 μm is composed of coarse, thick, plate-shaped calcite crystals, radially arranged, with milky-white color in plain-polarized light, and with dark cross in crossed polarized light,
- inner layer, with thickness ranging from 1.5 to 4.5 μm, is built of short fibrous calcite crystals chaotically oriented to the cyst surface, with yellowish, golden to brownish color in plain-polarized light, no dark cross is observed under the crossed-polarized light.
